# Retinoic acid signalling inhibits myogenesis by blocking MYOD translation in pig skeletal muscle cells

**DOI:** 10.1080/10495398.2024.2351973

**Published:** 2024-05-16

**Authors:** Changying Wang, Ruige Liu, Wenzhe Luo, Pengxiang Zhao, Heng Wang

**Affiliations:** a College of Animal Science and Technology, Shandong Agricultural University, Tai’an, China; b College of Animal Science and Technology, Huazhong Agricultural University, Wuhan, China

**Keywords:** Skeletal muscle stem cell, myogenic differentiation 1, pig, retinoic acid, retinoic acid receptor γ, vitamin A

## Abstract

Vitamin A is an essential nutrient in animals, playing important roles in animal health. In the pig industry, proper supplementation of vitamin A in the feed can improve pork production performance, while deficiency or excessive intake can lead to growth retardation or disease. However, the specific molecular mechanisms through which vitamin A operates on pig skeletal muscle growth as well as muscle stem cell function remain unexplored. Therefore, in this study, we isolated the pig primary skeletal muscle stem cells (pMuSCs) and treated with retinoic acid (RA), the natural metabolite of vitamin A, and then examined the myogenic capacity of pMuSCs via immunostaining, real-time PCR, CCK8 and western-blot analysis. Unexpectedly, the RA caused a significant decrease in the proliferation and differentiation of pMuSCs. Mechanistically, the RA addition induced the activation of retinoic acid receptor gamma (RARγ), which inhibited the myogenesis through the blockage of protein translation of the master myogenic regulator myogenic differentiation 1 gene (MYOD). Specifically, RARγ inactivate AKT kinase (AKT) signalling and lead to dephosphorylation of eukaryotic translation initiation factor 4E binding protein 1 (eIF4EBP1), which in turn repress the eukaryotic translation initiation factor 4E (eIF4E) complex and block mRNA translation of MYOD. Inhibition of AKT could rescue the myogenic defects of RA-treated pMuSCs. Our findings revealed that retinoid acid signalling inhibits the skeletal muscle stem cell proliferation and differentiation in pigs. Therefore, the vitamin A supplement in the feedstuff should be cautiously optimized to avoid the potential adverse consequences on muscle development associated with the excessive levels of retinoic acid.

## Introduction

Pork is a major source of meat protein for human and improving the lean meat production performance has always been an important research topic in pig genetics and breeding. Pork quantity and quality depend on various molecular and cellular processes that regulate porcine skeletal muscle cell proliferation and differentiation. Moreover, these molecular and cellular processes might be influenced by the epigenome comprising different mechanisms (e.g. DNA methylation, chromatin remodelling, histone tail modifications, microRNAs and non-coding RNAs) interact with environmental factors like nutrition, pathogens, climate to influence the expression profile of genes and the emergence of specific phenotypes.^1^^,^^2^ One of the basic research activities in domestic animals such as the pig is the study of genes and proteins related to economic traits and their regulation at the cellular or gene level by environmental and nutritional factors, and how to manage these factors to achieve efficient and optimal pork production.^3–5^

Vitamin A is essential for animal growth, reproduction, immunity and antioxidant function.^6^^,^^7^ Previous studies have shown that vitamin A supplementation can improve muscle and fat development in cattle and sheep embryos, by increasing the expression of genes and proteins related to myogenesis and myofibre types.^8^ Injection with vitamin A to newborn calves promoted myogenesis and muscle growth through increased satellite cell density and elevated proportions of oxidized type I and IIA myofibers.^9^ Neonatal intramuscular vitamin A injection upregulated paired box 7 (PAX7) expression and promoted the myogenic potential of satellite cells, which had a long-term effect of promoting the muscle growth of lambs.^10^ In chicken, dietary vitamin A can improve chicken growth performance, and maternal consumption of the vitamin A can also improve the body weight of offspring at hatching.^11^ Moreover, vitamin A can reduce diarrhoea, enhance lactogenic immune protection, and improve piglet growth performance significantly.^12^^,^^13^ Vitamin A also had a positive effect on bone health, as it can increase bone density and reduce the risk of fracture.^14^^,^^15^

Reasonable intake of vitamin A has a positive effect on muscle growth, bone development and immune capacity in animals. However, the mammals cannot synthesize vitamin A themselves, it must be supplied through continuous feed. Vitamin A insufficiency can affect the nutritional composition of meat products, carcass characteristics, and other meat quality features.^16^ In broilers, vitamin A deficiency results in darker meat colour and reduced water retention in the breast muscle, which in turn compromise meat quality. In pigs, vitamin A deficiency can cause severe diarrhoea and increased mortality.^11^^,^^17^

Although vitamin A is essential for the growth and development of animals, it should be added appropriately to avoid negative effects. Multiple studies have documented the negative effects of excessive vitamin A supplementation. For instance, the excessive amount of vitamin A intake could reduce the growth rate of finishing steers and the deposition of marbling.^18^ Pigs overdosed with vitamin A for a period of time have significantly deformed limbs.^19^ Intriguingly, long-term vitamin A restriction has been found to have a positive effect on the nutritional and sensory parameters of ham meat in Iberian pigs, ultimately improving meat quality.^20^ Previous studies have also shown that retinoic acid (RA), the natural metabolite of vitamin A, is essential for the myogenic function and differentiation of chicken limb muscles. But the excessive RA signalling inhibits myogenic differentiation, suggesting that the concentration of RA during limb myogenesis is complex and sensitive.^21^ Additionally, RA has ambivalent effects on the expression of myogenic differentiation 1 gene (MYOD) in myogenic cells. At lower concentrations (0.1–10 nM), RA significantly increases MYOD expression in muscle precursor cells, but at higher concentrations (0.1–1 μM), it inhibits MYOD expression.^22^ Therefore, it is crucial to understand the effects of supplemental vitamin A on animal development and health in order to formulate balanced feedstuff and achieve optimal performance in practical circumstances.

The biological effects of vitamin A are mainly mediated by the active metabolite RA.^23^^,^^24^ However, the molecular mechanisms of vitamin A action on porcine muscle growth are still unknown. In this study, we investigated the effects of vitamin A and its receptor retinoic acid receptor gamma (RARγ) on the proliferation and differentiation of pig primary skeletal muscle stem cells (pMuSCs) for the first time. Our findings provide new insights into the role of vitamin A in porcine muscle growth and suggest potential targets for improving meat production.

## Materials and methods

### Porcine primary muscle stem cells isolation

All animal procedures were performed according to the protocols of the Huazhong Agricultural University and the Institutional Animal Care and Use Committee (Hubei Province, China).

Porcine MuSCs were isolated according to the protocol described previously with slightly modification. In brief, one-day-old Yorkshire pig hind limb muscle was collected and the connective tissue was removed as much as possible with scissors. The muscle bundles were minced and digested with collagenase II (500 U/mL) in Dulbecco’s modified Eagle’s medium (DMEM, Gibco, Grand Island, New York, USA) for 90 min in a horizontal shaking water bath at 37 °C. Digest suspension was diluted with DMEM and then filtered with 40-μm cell strainers. The suspension was centrifuged at 500 *g* for 10 min at 4 °C. The pellet was resuspended and centrifuged at 600 *g* for 10 min at 4 °C for three times. Re-suspended cell pellets with skeletal muscle stem cell proliferation medium containing 20% foetal bovine serum (FBS, WenRen, Shanghai, China), 0.5% chicken embryo extract (CEE, Genimi, USA), 1% GlutaMax (Gibco, Grand Island, NY, USA), 1% Minimum Essential Medium Non-Essential Amino Acids Solution (MEM-NEAA, Gibco, Grand Island, NY, USA), 1% penicillin–streptomycin (Gibco, Grand Island, NY, USA), 2.5 ng/ml basic fibroblast growth factor (bFGF, Gibco, Grand Island, NY, USA), Roswell Park Memorial Institute 1640 medium (RPMI 1640, Gibco, Grand Island, NY, USA). The cell suspensions were seeded on culture dishes without coating and maintained at 37 °C in 5% CO_2_. After 2 h, the supernatant containing un-attached cells were then transferred to Matrigel-coated (Corning, USA) dishes and incubated for 24 h at 37 °C in 5% CO_2_. The culture dishes were washed three times with preheated phosphate buffered saline (PBS, Gibco, Grand Island, NY, USA) and replaced with proliferation medium on the next day.

### Cell culture and transfection

Porcine MuSCs were cultured on Matrigel-coated dishes with proliferation medium at 37 °C in 5% CO_2_. The cells were induced to differentiation using DMEM supplemented with 5% Horse Serum (HS, Gibco, Grand Island, New York, USA) at 37 °C in 5% CO_2_ when cell confluence reaches 80%-90%. Cells were transfected with 1 μg plasmid per well (12-well culture plate) using jetPRIME Transfection Reagent (Polyplus, Strasbourg, France) according to the user manual. After incubating for 4-6 h, cells were washed twice with preheated PBS and cultured in proliferation medium.

### Immunofluorescence staining

Cells were fixed with 4% paraformaldehyde (PFA, Biosharp, China) for 10 min, and then permeated with 0.5% Triton X-100 for 15 min. After blocking cells with blocking solution (0.5% Triton X-100 and 10% goat serum in PBS), cells were incubated with primary antibodies at 4 °C for overnight. Cells were washed with PBST (0.1% Triton X-100 in PBS) for three times and incubated with fluorophore-labelled secondary antibodies at room temperature for 2 h, washed and counterstained with 4′,6-diamidino-2-phenylindole (DAPI, Sigma-Aldrich, St. Louis, MO, USA). The differentiation index was calculated by the number of MYHC-positive nuclei/total nuclei.

### Antibodies

Antibodies used in this study are: anti-PAX7 (DSHB, IA, USA), anti-MF20 (MYHC, DSHB, IA, USA), anti-AKT (Proteintech, Chicago, USA), anti-phosphorylated-AKT (Cell Signaling Technology, Danvers, MA, USA), anti-eIF4EBP1 (ZENBIO, Morrisville, NC, USA), anti-phosphorylated-eIF4EBP1 (Cell Signaling Technology, Danvers, MA, USA), anti-MYOD (Proteintech, Chicago, USA), anti-myogenin (MYOG, Santa Cruz Biotechnology, Inc. Heidelberg, Germany), anti-β-Actin (ACTB, Abclonal, Wuhan, Hubei, China), anti-mouse secondary antibody conjugated with Alexa Fluor 555 (Invitrogen, Carlsbad, CA, USA), HRP anti-mouse secondary antibody (Beyotime, Shanghai, China), HRP anti-rabbit secondary antibody (Beyotime, Shanghai, China).

#### EdU incorporation analysis

5-ethynyl-2′-deoxyuridine (EdU, Invitrogen, Carlsbad, CA, USA) was dissolved in sterilized PBS at 2.5 mg/mL and stored at −20 °C. The stock EdU solution was diluted to 0.5 mg/mL with PBS. EdU was dissolved in culture medium 2 h before analysis. Cultured cells were fixed with 4% PFA for 10 min, and then permeated with 0.5% Triton X-100 for 15 min. After washed with PBS for three times, cells were incubated with EdU staining buffer (100 mM Tris, 1 mM CuSO_4_, 10 mM fluorescent azide, and 100 mM ascorbic acid for 30 min at room temperature as previously described.^25^ Stained cultured cells were counterstained with DAPI. The ratio of EdU positive cells in images were analysed with ImageJ software (version 1.8.0) and plotted using Graphpad software (version 8.0).

#### Western blot

Cells were rinsed twice with pre-chilled PBS. To prepare cell lysates, cells were scraped and lysed using RIPA lysis buffer (Beyotime, Shanghai, China) with phenylmethanesulfonyl fluoride (PMSF, Beyotime, Shanghai, China) and Sodium Orthovanadate (Beyotime, Shanghai, China) on ice for 30 min. The lysate was centrifuged at 13000 rpm at 4 °C for 15 min and then 5 × protein loading buffer (Epizyme, Shanghai, China) was added to the lysates prior to their full denaturation in 100 °C heating blocks for 10 min. Protein concentration was measured by BCA Protein Assay Kit (Beyotime, Shanghai, China) according to the user manual. A total of 30 μg of protein was electrophoresed on SDS/PAGE gel and transferred to a polyvinylidene difluoride (PVDF) membrane (Millipore, Billerica, MA, USA). The membranes were blocked in 5% bovine serum albumin for 2 h and then incubated with primary antibodies at 4 °C overnight. The membranes were washed with 0.5% TBS-Tween 20 and then incubated with goat anti-mouse or anti-rabbit secondary antibodies conjugated with western chemiluminescent HRP substrate for 2 h. The membranes were washed and exposed using a GE LAS 4000 imaging system (GE, USA). The captured images were analysed using ImageJ software. The protein level of whole cell lysates normalized against the expression of ACTB.

#### Real-time PCR

RNA was extracted from cells of different treatments, and the 500–1000 ng of RNA was reverse transcribed to single-stranded cDNA using an RT kit after passing a quality test (Novoprotein, Suzhou, Jiangsu, China), involving two steps as follows: 42 °C for 5 min, followed by 37 °C for 15 min. The qPCR was performed using a Hieff qPCR SYBR Green MIX (Yeasen, Shanghai, China), in triplicate, on a Bio-Rad Touch CFX384 (Bio-Rad, Hercules, California, USA). The qPCR program was as follows: 95 °C for 30 s, followed by 40 cycles of 5 s at 95 °C and 40 s at 60 °C. Primer sequences for all qPCR are detailed in Supplementary Data S^2^. The 2^-ΔΔCT^ algorithm was employed to estimate the relative expression level of each gene on Excel software.

### Expression vector construction

To create RARγ expression vector, RARγ coding sequences was amplified and cloned into pEGFP-C1 using restriction enzyme of *NheI* and *BamHI* (Thermo Scientific, Waltham, MA, USA) to replace EGFP coding sequence. Related primers for the RARγ expression vector were designed in reference to the Ensembl database (ENSSSCT00045003481.1) and were shown in Supplementary Data S^3^. Homologous recombination of RARγ PCR fragments and vector plasmid were performed using homologous recombination mix (Applied Biological Materials Inc., Richmond, Canada) according to the user manual. Recombinant expression vectors were transformed into Fast T1 competent cells (Vazyme, Nanjing, Jiangsu, China) and plated on agarose Luria-Bertani (LB) medium overnight. Bacteria clones were cultured in liquid LB medium for 4-6 h and genotyped with PCR. Sequences of expression vectors were validated by Sanger sequencing. Plasmids were extracted using E.Z.N.A. Endo-Free Plasmid Mini Kit II (OMEGA, Suzhou, Jiangsu, China) according to the user manual.

### Cell treatment and drugs

The pMuSCs were treated with gradient concentrations of RA (10^−9^ M-10^−3^ M) (Sigma- Aldrich, St. Louis, MO, USA) or 0.1% Dimethyl sulfoxide (DMSO, Solarbio, Beijing, China) in proliferation medium. EdU staining performed 36 h or 48 h after RA treatment to detect cell proliferation efficiency. Western blot was performed 48 h after different treatments. Immunofluorescence staining was performed to assess differentiation ability after 48 h of incubation in differentiation medium.

For pMuSCs administrated with drugs and expression plasmids, pMuSCs were pre-cultured with proliferation medium in the presence of 1 μM RA or 0.1% DMSO for 24 h before transfection. EdU staining and Western blot were performed 48 h after transfection. Immunofluorescence staining was performed to assess differentiation ability after 48 h of incubation in differentiation medium.

For RARγ rescue experiments, pMuSCs were pre-cultured for 24 h before transfection with growth medium in the presence of 1 μM RA or 0.1% DMSO. Then 1 μM BMS493 (MCE, New Jersey, USA) was added to the medium 4 h after the cells were transfected with RARγ overexpression plasmid. EdU staining was performed 48 h after transfection. At the same time, the remaining cells started to induce differentiation, and the differentiation ability was detected by immunofluorescence after 48 h.

For AKT rescue experiments, pMuSCs were pre-cultured for 24 h before transfection with growth medium in the presence of 1 μM RA or 0.1% DMSO. Then 10 μM SC79 (MCE, New Jersey, USA) was added to the medium 4 h after the cells were transfected with RARγ overexpression plasmid. EdU staining was performed 48 h after transfection. At the same time, the remaining cells started to induce differentiation, and the differentiation ability was detected by immunofluorescence after 48 h.

### Quantification and statistical analysis

Real-time quantitative polymerase chain reaction (RT-qPCR) results were presented as mean ± SEM. Statistical comparison between two groups was performed by two-sided unpaired Student’s *t*-test. Multi-group comparisons were performed with one-way ANOVA test followed by Bonferroni post hoc test using GraphPad Prism (GraphPad Software, Inc.). Immunofluorescence analysis was shown as mean ± SEM after nucleus number statistics analysis with ImageJ software (National Institute of Mental Health, Bethesda, MD, USA). In all figures, asterisks denote statistical significance.ns: not significant, *P < 0.05, **P < 0.01, ***P < 0.001, ****P < 0.0001.

## Results

### Retinoic acid inhibited pMuSCs proliferation and differentiation

**Figure 1.**
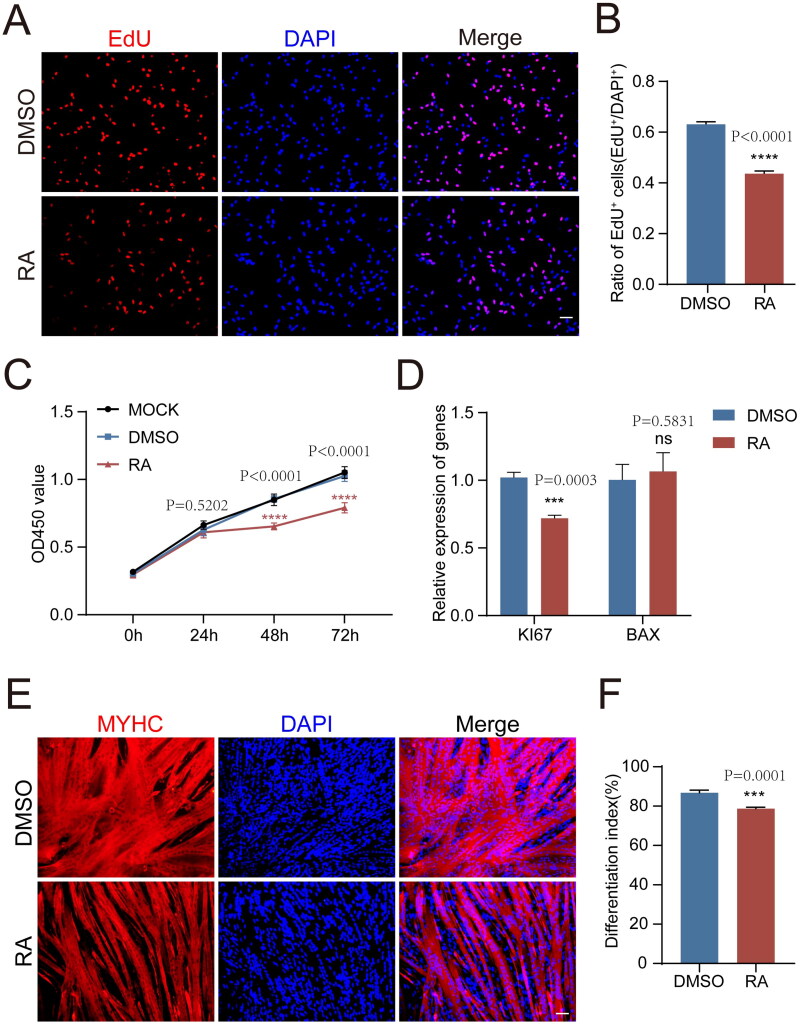
The proliferation and differentiation properties of pMuSCs were inhibited by the addition of RA. A: Representative pictures showing the EdU assay in 1μM RA-treated pMuSCs for 48 h, (Scale bars: 50 µm). B: Quantification of EdU-positive pMuSCs in B. n= 3 independent assays/condition, >3000 cells counted/assay. C: CCK8 assay result showing the effect of RA on pMuSCs proliferation compared to that of control cells treated with 0.1%DMSO. D: The mRNA expression levels of *KI67* and *BAX* after RA treatment in pMuSCs for 48 h. E: MYHC staining of pMuSCs after addition of RA and induction of differentiation for 48 h (Scale bars: 50 µm). F: Quantification of the differentiation index in E.

Muscle stem cells proliferation and differentiation to constitute the myofibers, which are essential for the growth and development of skeletal muscle tissue.^26^ To investigate the potential role of vitamin A in pig myogenesis, we isolated pMuSCs and treated the cells with RA. Firstly, we treated cells with gradient concentrations of RA (10^−9^M-10^−3^M) for 36 h and examined the proliferation ability of the cells. We found that both high and low concentrations of RA inhibited the proliferation efficiency of pMuSCs (Fig. S1). In the subsequent experiments, the cells were treated with RA at a concentration of 1 μM by default unless otherwise specified since 1 µM of RA is the suitable concentration for efficiently inhibiting cell proliferation and ensuring optimal cell status simultaneously. The EdU assay showed that the proportion of proliferating pMuSCs decreased from an average of 63% in the control group to 43% after RA treatment for 48 h (*P* < 0.0001) (Fig. 1A and B). CCK-8 assay also showed that RA significantly inhibited cell proliferation (*P* < 0.0001) (Fig. 1C). The expression of cell proliferation marker gene *KI67* was also significantly decreased in the RA group (*P* < 0.0001), while the expression of apoptosis gene *BAX* was not changed (*P* = 0.5813) (Fig. 1D). It indicated that the reduced cell numbers were indeed caused by impaired proliferation but not the cell death. In addition, the differentiation capacity of the pMuSCs was severely impaired by RA treatment, as indicated by a significant decrease in the differentiation index (*P* = 0.0001) (Fig. 1E and F). Therefore, exogenous RA supplementation could inhibit the proliferation and differentiation of pMuSCs, thus impairing the myogenic process.

### RA-induced RARγ further suppressed the myogenic capacity of pMuSCs

**Figure 2.**
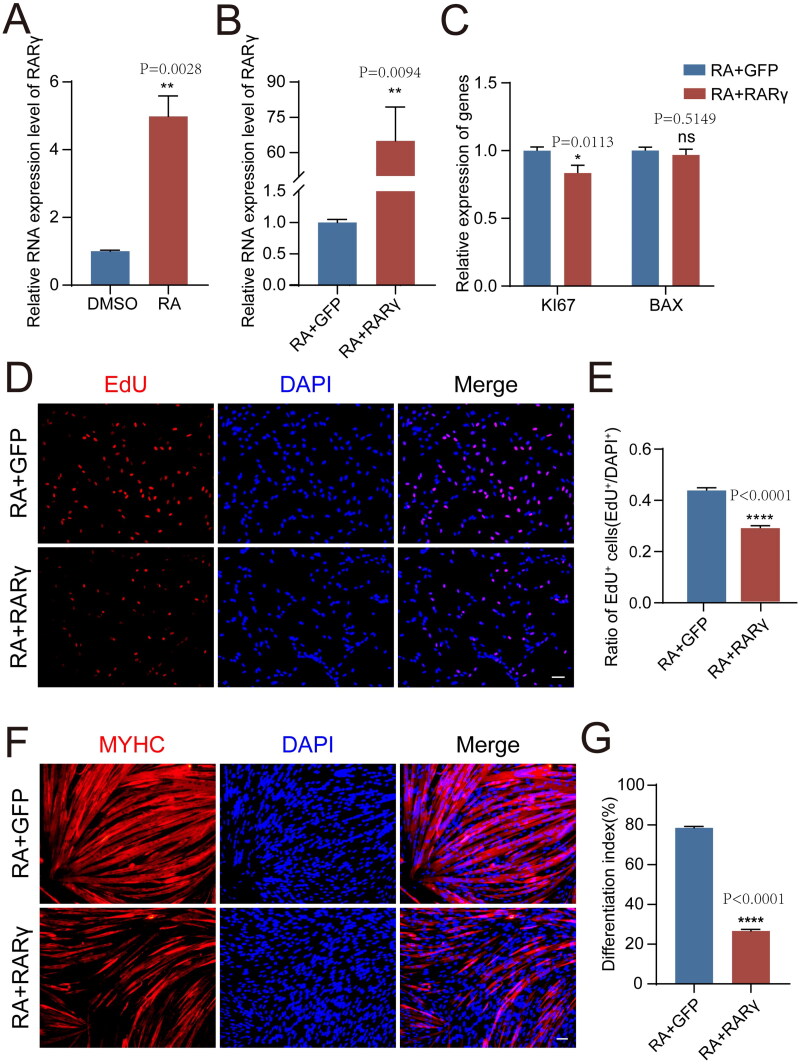
RA and RARγ further inhibited the proliferation and differentiation ability of pMuSCs. A: The mRNA expression levels of *RARγ* after RA treatment in pMuSCs for 48 h. B: The efficiency of *RARγ* overexpression in pMuSCs was detected by qPCR. C: The mRNA expression levels of *KI67* and *BAX* of RA-treated pMuSCs after overexpression *RARγ* for 48 h. D: The proliferation ability of RA-treated pMuSCs was detected by EdU staining after overexpression *RARγ* for 48 h (Scale bars: 50 µm). E: Quantification of EdU-positive pMuSCs. n= 3 independent assays/condition, >3000 cells counted/assay. F: MYHC staining of RA-treated pMuSCs overexpressed the *GFP* and *RARγ* respectively and induced differentiation for 48 h (Scale bars: 50 µm). G: Quantification of the fusion index in F.

Previous research has demonstrated that RA acts as a ligand to assemble complexes with nuclear receptors that bind to DNA elements and activate the expression of target genes.^27^ Accordingly, we found that the expression of *RARγ* was significantly elevated in RA-treated pMuSCs compared to control cells treated with DMSO (*P* = 0.0028) (Fig. 2A), indicating that RA treatment indeed induced the expression of the related receptor. Correspondingly, the overexpression of *RARγ* in the presence of RA could further inhibit the cell proliferation and differentiation compared to the RA treatment alone. RT-qPCR result showed that the expression of cell proliferation marker gene *KI67* was significantly decreased in the RA + RARγ group compared to RA + GFP group (*P* = 0.0113), while the expression of apoptosis gene *BAX* was not changed (*P* = 0.5813) (Fig. 2C). And the proportion of proliferating pMuSCs in RA + RARγ treatment group further declined to 28% from 43% in RA + GFP- treated cells (*P* < 0.0001) (Fig. 2D and E). And the differentiation index also dropped to 27% after RA + RARγ treatment from 79% in the RA + GFP-treated cells (*P* < 0.0001) (Fig. 2F and G). The overexpression efficiency of *RARγ* was validated using RT-qPCR (Fig. 2B). Collectively, these findings implied that RA-mediated RARγ signalling further suppressed both proliferation and differentiation of pMuSCs.

To further verify that RA signalling via the actions of *RARγ* to block the cell proliferation and differentiation, we performed rescue experiments by inhibiting **RARγ** activity using BMS493, a pan-antagonist against the RAR.^28^ We treated pMuSCs with RA + RARγ and BMS493 and then examined whether cell proliferation and differentiation capacities could be restored via blocking **RARγ**. As expected, the proliferation and differentiation capacity of pMuSCs was elevated to comparable levels with the control after the addition of BMS493 (Fig. S2). Collectively, these results confirmed that *RARγ* is a vital receptor activated by RA that inhibit the proliferation and differentiation capacity of pMuSCs in conjunction with RA.

### Retinoic acid and RARγ inhibit MYOD protein synthesis

**Figure 3.**
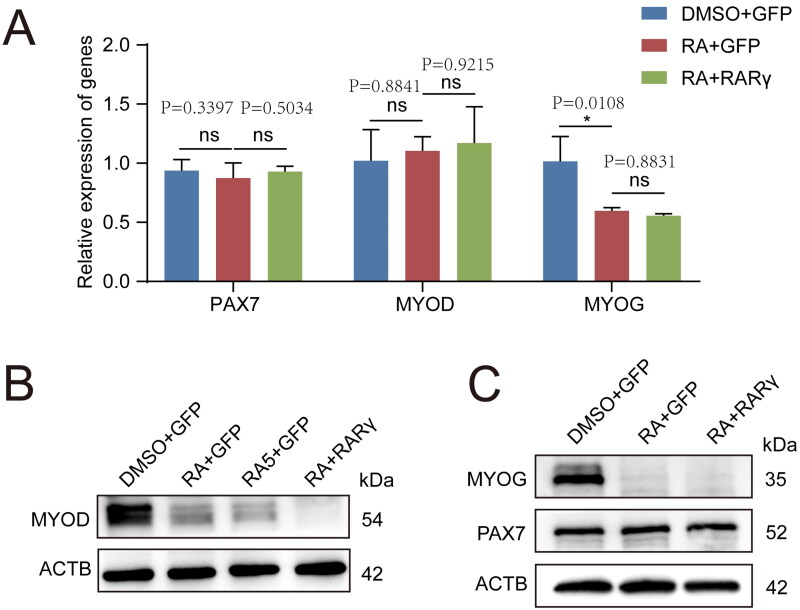
The RARγ signal which was activated by RA repressed **MYOD** protein translation in pMuSCs. A: The mRNA expression levels of *PAX7*, *MYOD* and *MYOG* after different treatments, including DMSO + GFP, RA + GFP and RA + RARγ. B: Western blot analysis of the protein expression levels for **MYOD** after different treatments, including DMSO + GFP, RA + GFP, RA5 + GFP and RA + RARγ. **ACTB** was used as the control. RA5 represents the treatment concentration of RA is 5 μM, RA represents the treatment concentration of RA is 1 μM. C: Western blot analysis of the protein expression levels for **PAX7** and **MYOG** after different treatments, including DMSO + GFP, RA + GFP and RA + RARγ. **ACTB** was used as the control.

To further investigate the specific pathways underlying RA/RARγ effects in pMuSCs, we performed RT-qPCR to analyse the expression levels of classical marker genes involved in the proliferation (*MYOD, PAX7*) and differentiation (*MYOG*) upon RA + RARγ treatment. Surprisingly, the mRNA expression of *MYOD* or *PAX7* showed no difference between the cells treated with RA + GFP (*P* = 0.8841) and the control cells treated with DMSO + GFP (Fig. 3A). There was also no difference in *MYOD* or *PAX7* mRNA expression between RA + GFP-treated cells and RA + RARγ-treated cells (*P* = 0.9125) (Fig. 3A). However, the mRNA expression of *MYOG*, a late differentiation marker gene whose transcriptional regulatory region is the direct target of **MYOD** protein, was dramatically downregulated when pMuSCs were treated with RA + GFP (*P* = 0.0108) and further decreased when treated with RA + RARγ (Fig. 3A). Correspondingly, Western blot showed that the **MYOG** protein was decreased upon RA + GFP treatment compared to the control cells treated with DMSO + GFP, and further deceased when cells treated with RA + RARγ (Fig. 3C). Since *MYOG* is known to be a canonical direct transcriptional target of **MYOD** and reflects **MYOD** protein activity, we examined the protein levels of **MYOD.**^29^^,^^30^ Western blot analysis revealed that the protein abundance of **MYOD** was reduced by treatment with RA + GFP or RA + RARγ compared to the cells treated with DMSO + GFP(Fig. 3B), while the protein level of **PAX7** remained unchanged (Fig. 3C). The Western blot results in Fig. 3B also showed that the protein abundance of **MYOD** in the RA + RARγ treatment group decreased more obviously than that in the RA5 + GFP treatment group (i.e., 5 μM-RA + GFP) compared to the cells treated with DMSO + GFP, indicated that the combination of RA and RARγ produced a more effective inhibitory effect than increasing the concentration of RA alone, and further clarified the importance of *RARγ*. These results suggested that *MYOD* translation, rather than *MYOD* transcription, was responsible for the decline of cell proliferation and differentiation. Moreover, RA-activated RARγ signalling played a role in regulating *MYOD* translational repression in pMuSCs, which controlled myoblast differentiation.

### Retinoic acid and RARγ affects MYOD protein synthesis via the AKT/eIF4EBP1 signaling cascade

**Figure 4.**
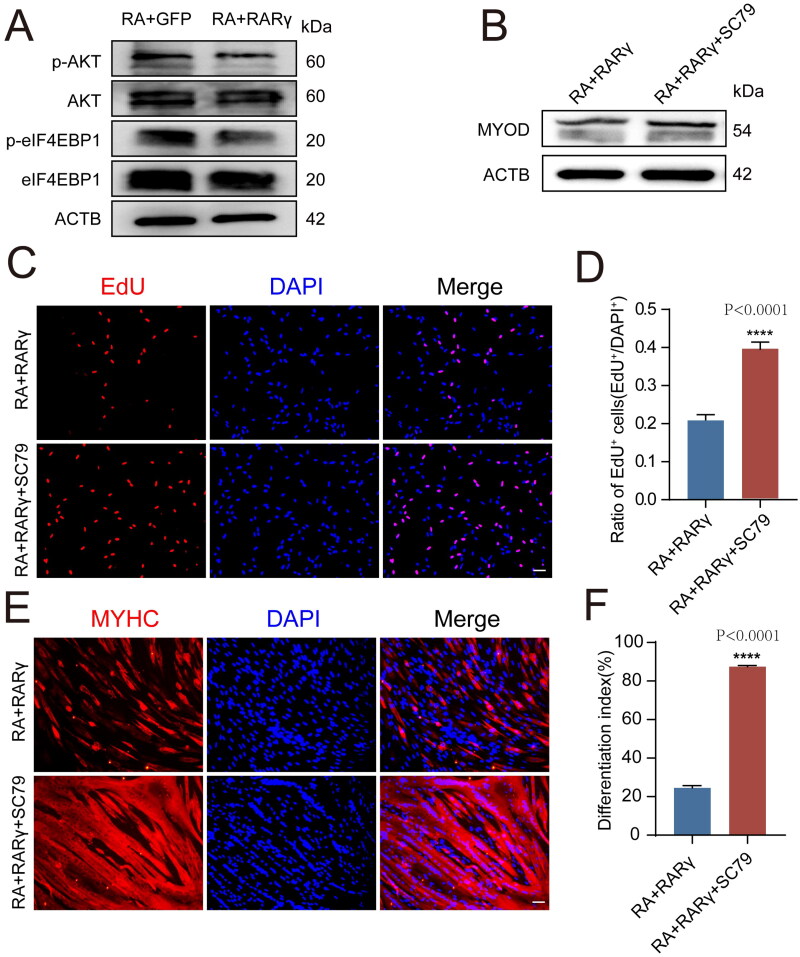
RARγ affects **MYOD** protein synthesis via the AKT/eIF4EBP1 signalling cascade. A: Western blot analysis of protein levels of **p-AKT**, **AKT**, **p-eIF4EBP1**, **eIF4EBP1** and **ACTB** after different treatments, including RA + GFP and RA + RARγ. B: Western blot analysis of protein levels for **MYOD** in RA + RARγ group and RA + RARγ + SC79 group treated pMuSCs. C: The EdU staining of RA + RARγ group and RA + RARγ + SC79 group treated pMuSCs (Scale bars: 50 µm). D: Quantification of EdU-positive pMuSCs. n= 3 independent assays/condition, >3000 cells counted/assay. E: MYHC staining of RA + RARγ group and RA + RARγ + SC79 group treated pMuSCs after induced differentiation for 48 h (Scale bars: 50 µm). F: Quantification of the fusion index in E.

Although RA-activated *RARγ* decreased the expression of **MYOD** protein, the mechanism by which RA or *RARγ* blocked the synthesis of **MYOD** protein in pMuSCs was still unclear. Previous studies have shown that RA can inhibit **AKT** activity, which stimulates mRNA translation by phosphorylating the translation initiation repressor **eIF4EBP1.**^31^^,^^32^ In particular, only dephosphorylated **eIF4EBP1** can interact with eukaryotic translation initiation factor 4E (**eIF4E**) to inhibit the assembly of the translation initiation complex, and the phosphorylation of **eIF4EBP1** results in its dissociation from **eIF4E** and mRNA translation initiation.^33^ Therefore, we postulated that RA + RARγ could inactivate **AKT** and dephosphorylate **eIF4EBP1** to inhibit translation initiation of *MYOD* mRNA. In order to validate the hypothesis, we treated pMuSCs with RA accompanied by overexpressing *RARγ*, and then analysed the phosphorylation levels of **AKT** and **eIF4EBP1** by immunoblotting. As expected, Western blot results revealed that both **AKT** and **eIF4EBP1** phosphorylation levels were decreased in RA + RARγ treated cells compared to cells treated RA + GFP (Fig. 4A).

To further verify that **AKT** is the main effector regulated by *RARγ* that control the **eIF4EBP1** phosphorylation and **MYOD** protein synthesis, we performed a rescue experiment by manipulating **AKT** activity. We treated RA + RARγ administrated pMuSCs with an **AKT** activator SC79 and examined the levels of **MYOD** protein expression. Western blot analysis showed that in RA + RARγ-treated cells, the constitutive activation of **AKT** with SC79 increased **MYOD** protein expression (Fig. 4B). These results confirmed that **AKT** is the major effector downstream of RA/RARγ signaling that regulates **MYOD** protein synthesis in pMuSCs. Next, we examined the cell cycle status upon **AKT** activation in RA + RARγ-treated pMuSCs. Accordingly, the **AKT** activator SC79 relieved the proliferation inhibitory caused by RA + RARγ treatment and stimulated cell proliferation (*P* < 0.0001) (Fig. 4C and D). Moreover, SC79 also alleviated the effect of RA + RARγ on blocking pMuSCs differentiation as shown by increased myotubes formation in RA + RARγ + SC79-treated cells compared to RA + RARγ-treated cells (*P* < 0.0001) (Fig. 4E and F).

We conclude that *RARγ* can inactivate **AKT**, leading to the dephosphorylation of **eIF4EBP1** and blockage of protein synthesis. Consequently, impaired **MYOD** protein synthesis restricts the cell proliferation and further myogenic differentiation of pMuSCs, resulting in a constant state of strong stemness and weak myogenesis.

## Discussion

The composition of pork, which is derived from pig muscle, is determined by a combination of muscle cell hypertrophy and hypotrophy. To achieve desired meat quality and yield in pork production, it is important to carefully manage factors that can influence muscle cell proliferation and differentiation, such as nutrition, genetics, and the interaction between them.^34^ Vitamin A is a key vitamin in pig feed. The plant feedstuffs used to compound swine feed do not contain relevant amounts of vitamin A. Therefore, the supplementation of synthetic vitamin A and its derivatives is essential to meet the growth requirement. A positive effect of optimal vitamin A supplementation on swine reproduction, performance, immunity, and health has been demonstrated. However, the specific molecular mechanisms through which vitamin A operates on the gene expressions in pig skeletal muscle cells have remained unclear.

Vitamin A mainly exerts its biological functions through the active metabolite RA. There are numerous studies on the role of vitamin A in lipid metabolism, and comparatively few studies related to its role in muscle growth and development, especially in the porcine.^35^^,^^36^ In zebrafish studies, RA was able to induce myogenic gene expression and muscle differentiation by mediating Fgf8 signaling.^37^ In beef cattle, the administration of vitamin A to newborn calves resulted in an increase in the number of muscle stem cells, which promoted myofiber fusion and a significant increase in muscle mass accompanied with weight gain.^9^^,^^38^ RA treatment in primary myoblasts from sheep similarly increased protein levels such as MYOG and MYHC, all of which suggest a critical role for RA in the myogenic lineage.^39^ This study aims to investigate if RA plays a similar role in pig muscle to explore potential species differences.

Muscle stem cells are an important component of skeletal muscle, which form mature myofibers after cell proliferation, differentiation and fusion to form new myofibers (myotubes), thus we could use the proliferation and differentiation of muscle stem cells *in vitro* to mimic the process of myogenesis. We selected pig muscle stem cells (pMuSCs) as a model to explore the role of vitamin A in porcine myogenesis. It has been shown that RA treatment causes cell proliferation arrest and morphological changes.^40^^,^^41^ In addition, RA exhibited an inhibitory effect on the differentiation process in both isolated cultured mouse muscle stem cells and human myoblasts.^42^^,^^43^ In the present study, we found that RA inhibited the proliferation and differentiation of pMuSCs and increased the stemness of pMuSCs, which was confirmed by staining with EdU and MYHC. Also, the retinoic acid receptor expression was correspondingly increased by RA treatment. The RA jointly regulated gene expression by binding with retinoic acid receptor RARγ, and when we further overexpressed RARγ on RA-treated cells, the proliferation and differentiation ability of the cells was further decreased.

MYOD is a marker of early myogenic differentiation and is also required for muscle differentiation during growth and regeneration.^44^,^45^ Although lower or absent of MYOD mRNA decreases its protein level and contribute to muscle stem cell immaturity, *in vivo* data demonstrated that quiescent pMuSCs transcribed MYOD mRNA but protein was absent.^46^^,^^47^ Translational or post-translational regulation of MYOD genes may be more compatible with *in vivo* stemness maintenance. Meanwhile MYOD-deficient muscle stem cells exhibit a significantly reduced differentiation capacity, so it is clear that MYOD controls the myogenic capacity of muscle stem cells and the level of its immaturity.^48^,^49^ RA and RARγ control muscle differentiation probably by mediating MYOD family to play a function. In pMuSCs, we determined that RA-activated RARγ reduced MYOD translation, blocked protein synthesis. MYOG, a downstream target of MYOD that is also required for late myogenesis, induces myoblast fusion into myofibres during terminal differentiation.^50^ When treated with RA + RARγ group, the protein level of MYOG was also decreased and the cell fusion ability became poor. In this study, RA + RARγ group treatment inhibited terminal differentiation and also inhibited myotubes fusion, suggesting that vitamin A affects the formation of mature myotubes, which is similar with the results of the inhibitory effect of vitamin D on myogenesis.^51^

Muscle mass changes are determined by factors such as protein synthesis and degradation, contributing to size changes in myofibers.^52^ For example, the mTOR signalling pathway, which affects protein synthesis, regulates several processes including translation initiation and elongation factors, and the biogenesis of the ribosomes themselves.^53^ The mTOR pathway has the most important role of AKT phosphorylation to promote cell survival, proliferation and growth.^54^^,^^55^ AKT phosphorylates p70S6K under the regulation of PI3K, which accelerates muscle protein synthesis, and by the induction of Foxo3a phosphorylation inhibits protein degradation in myogenic cells.^56^ Research has shown that RA can block AKT activity, stimulating mRNA translation by phosphorylating the translation initiation repressor eIF4EBP1.^31^^,^^44^ In this study, we found that RA + RARγ treatment of cells decreased AKT phosphorylated protein levels and eIF4EBP1 phosphorylated protein levels and blocked MYOD protein synthesis. The AKT activator SC79 treatment of RA + RARγ group cells promoted MYOD protein synthesis, rescued the cell proliferation inhibitory effect caused by RA + RARγ, it also significantly improved cell differentiation and formed more multinucleated myotubes, thereby enhancing myogenesis. This also suggests that vitamin A affects protein synthesis in myotubes through phosphorylation of AKT and eIF4EBP1.

**Figure 5.**
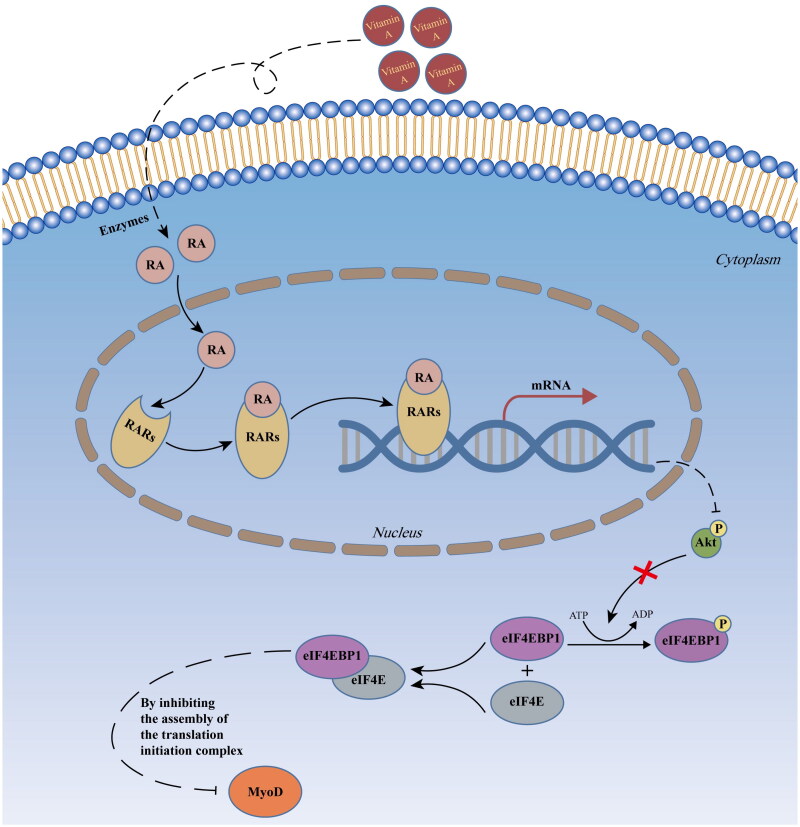
Schematic illustration of retinoic acid signaling inhibiting myogenesis by blocking *MYOD* translation in pig muscle stem cells.

In conclusion, we found that RA and RARγ hinder MYOD protein synthesis by attenuating the phosphorylation of AKT and eIF4EBP1, preventing the proliferation and differentiation of pMuSCs and preserving the stemness of pMuSCs (Fig. 5). This finding provides additional insight into the regulatory mechanisms of vitamin A in the maintenance of stemness of muscle cells as well as muscle growth in pigs. Current study also highlights that the optimal and strict levels of vitamin A supplement should be practiced to attain optimal performance of muscle growth in pigs. The reasonable addition of vitamin A to feedstuffs can not only improve pork production to a certain extent, but also help to save production costs.

## Supplementary Material

Supplemental Material

Supplemental Material

Supplemental Material

Supplemental Material
