# Supplementary material for: Retinoic acid signalling inhibits myogenesis by blocking MYOD translation in pig skeletal muscle cells
Source: Anim Biotechnol. 2024 May 16;35(1):2351973. doi: 10.1080/10495398.2024.2351973 (PMC12674307; doi:10.1080/10495398.2024.2351973)
Supplement: Supplemental Material [file LABT_A_2351973_SM4376.docx]

**Supplementary Data S3: Original images of all Western Blot images appearing in the article.**

| 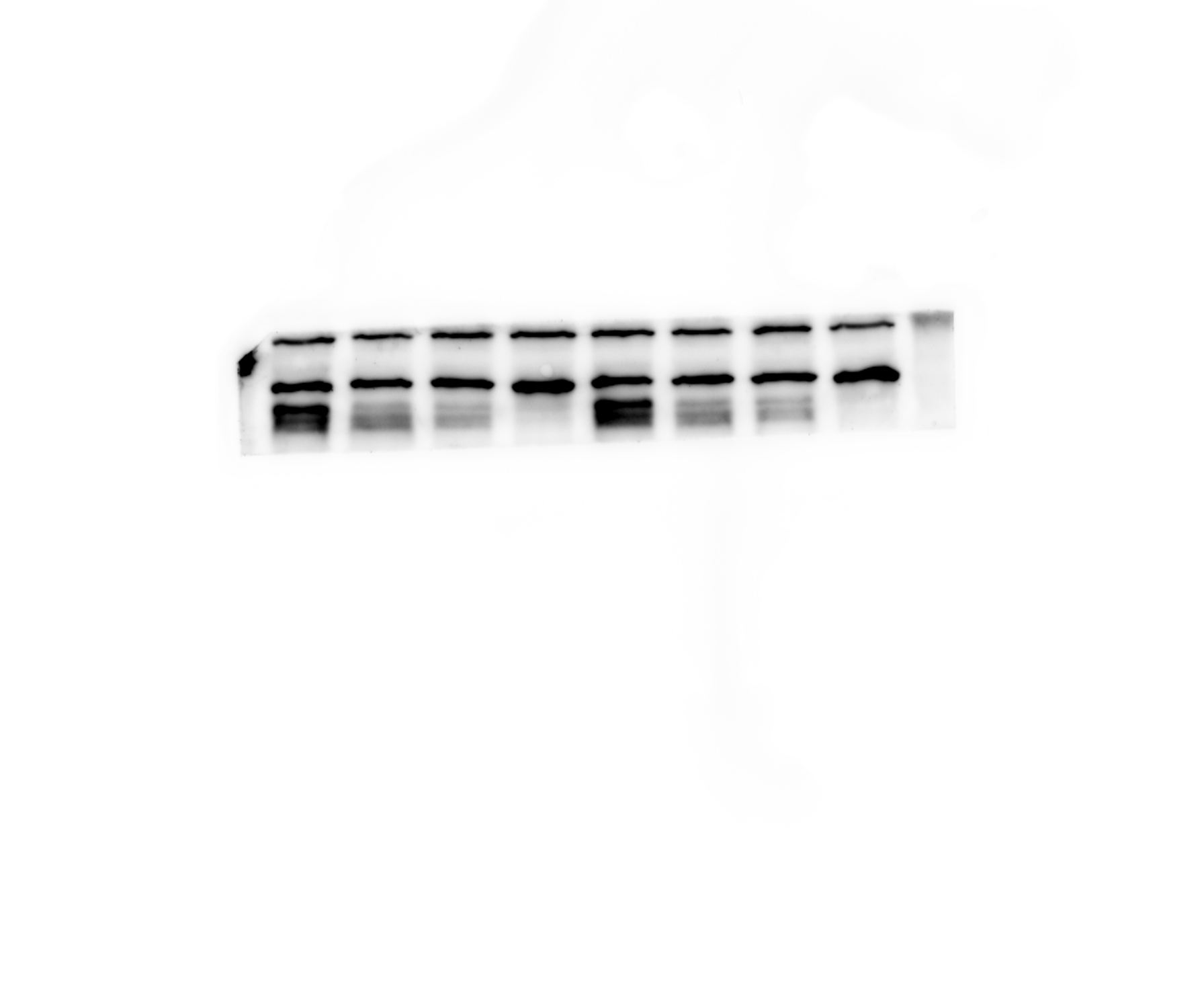 |
| --- |
| **Figure 3B**-**MYOD** |
| 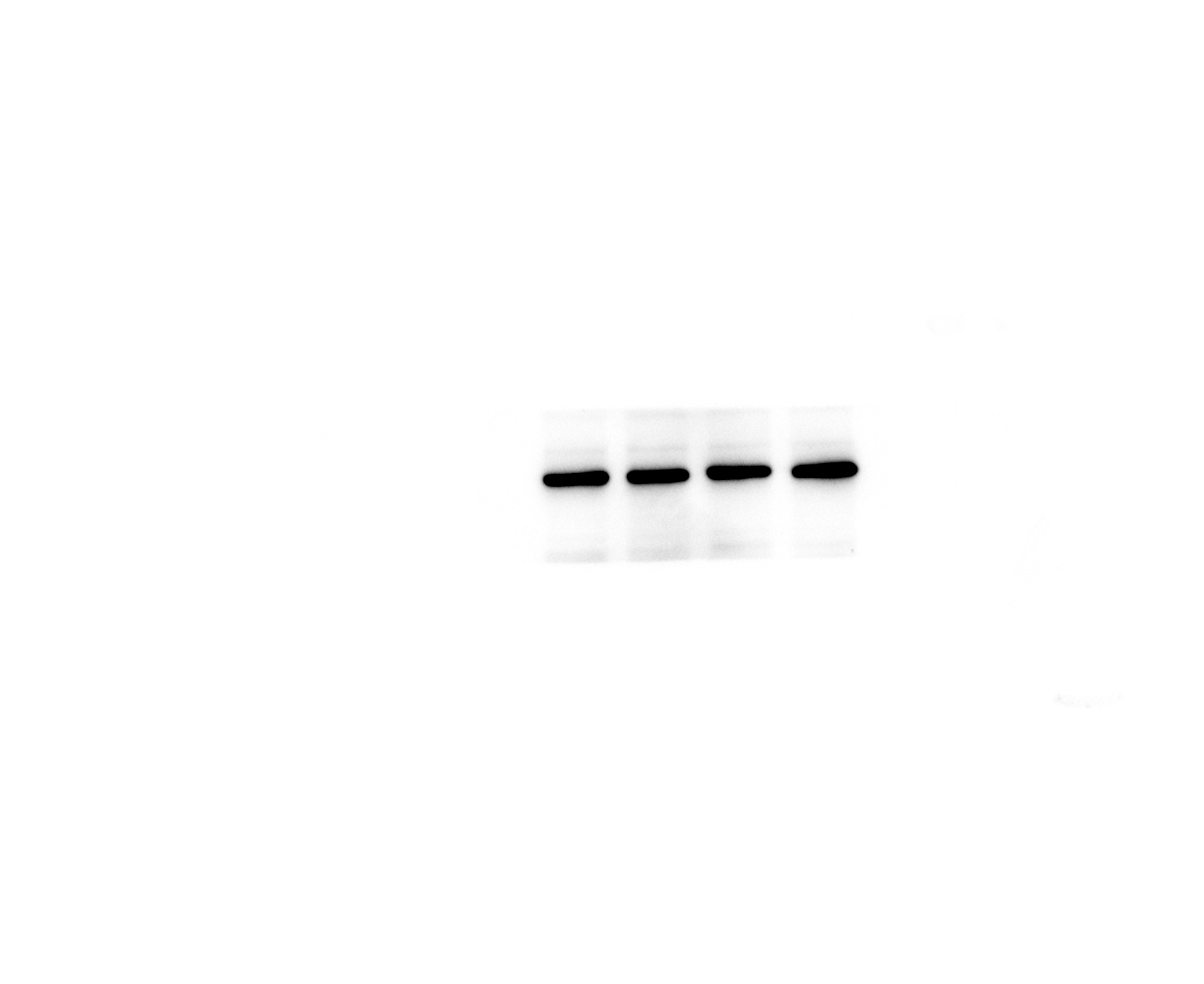 |
| **Figure 3B**-**ACTB** |
| 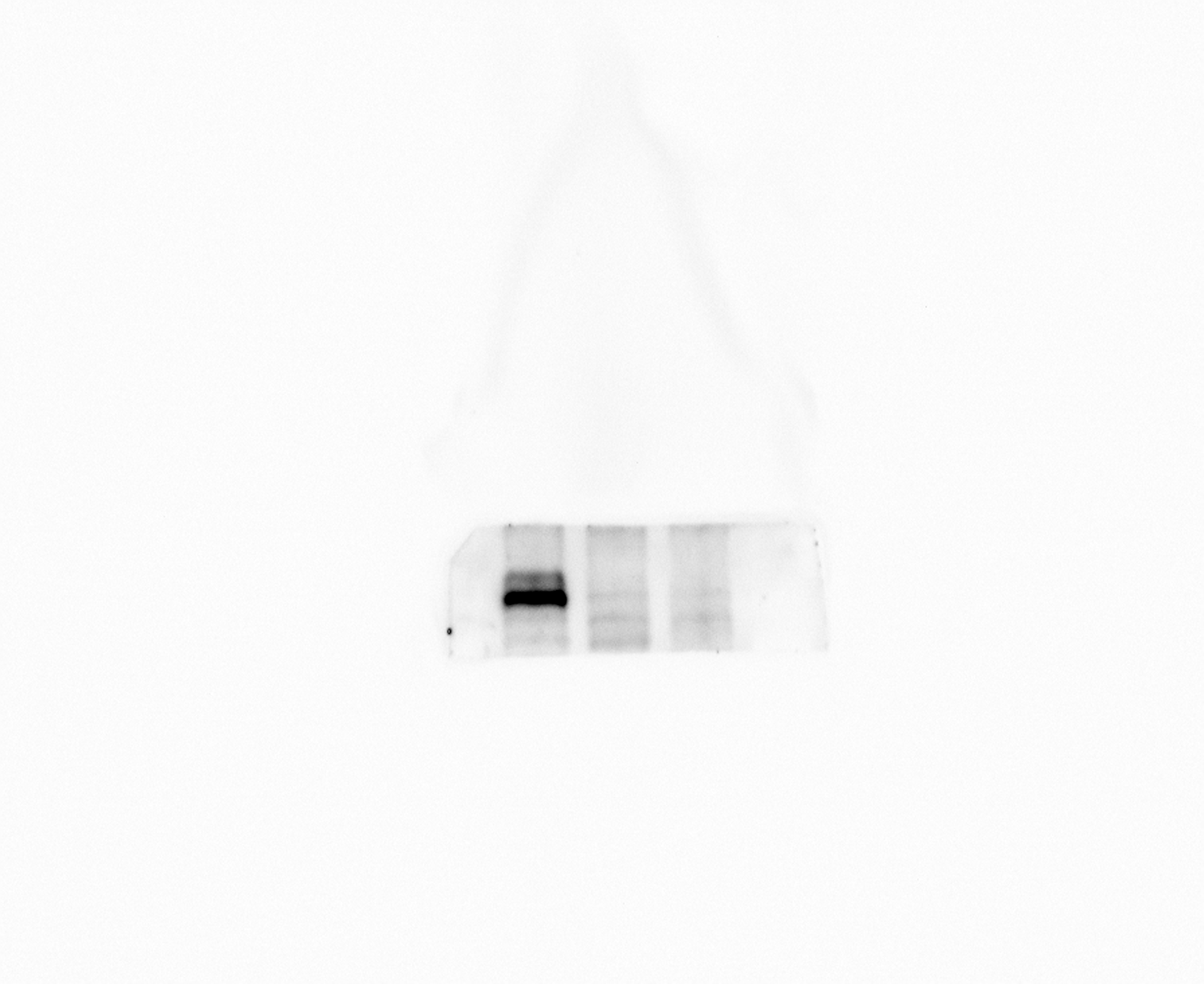 |
| **Figure 3C**-**MYOG** |
| 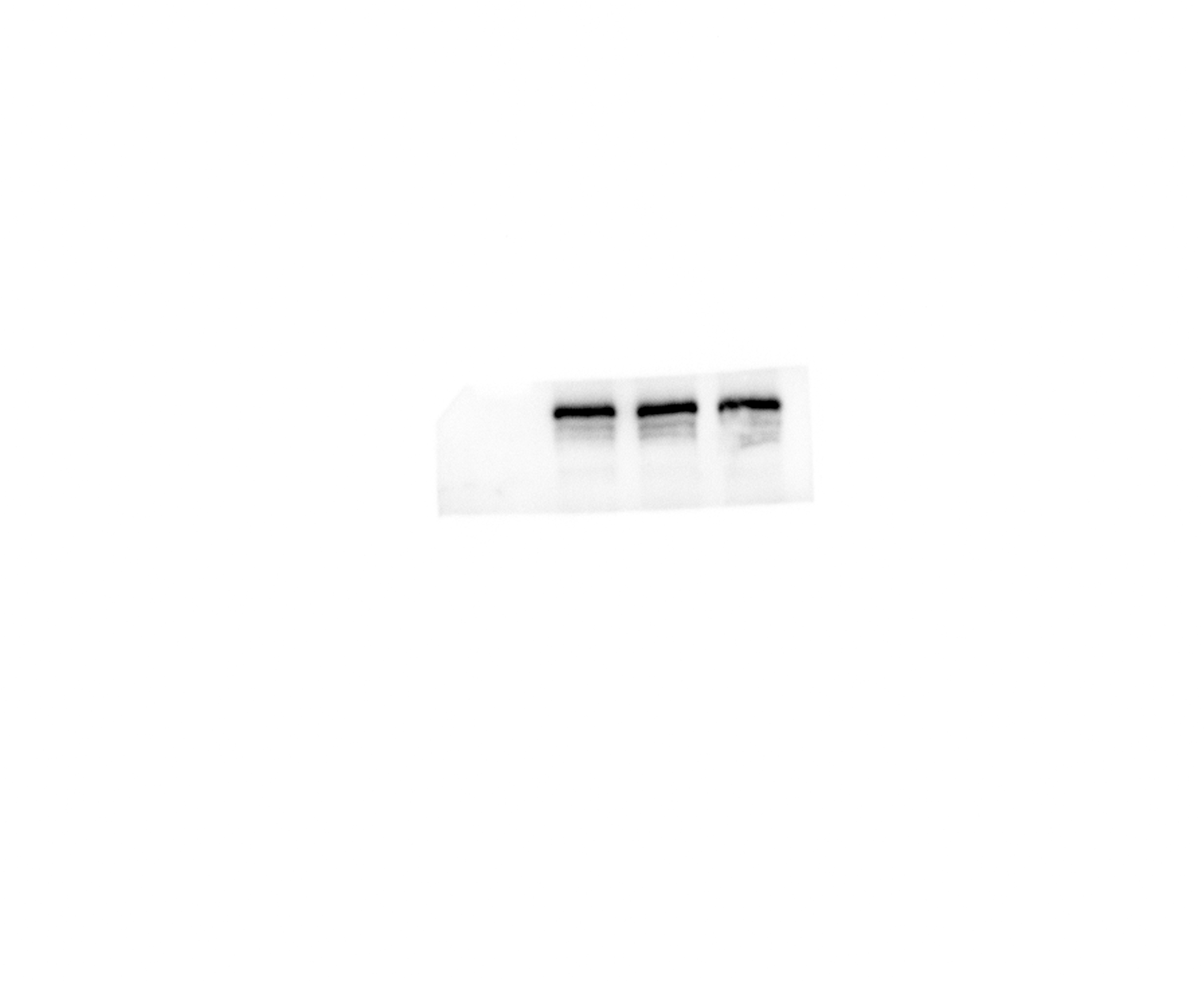 |
| **Figure 3C**-**PAX7** |
| 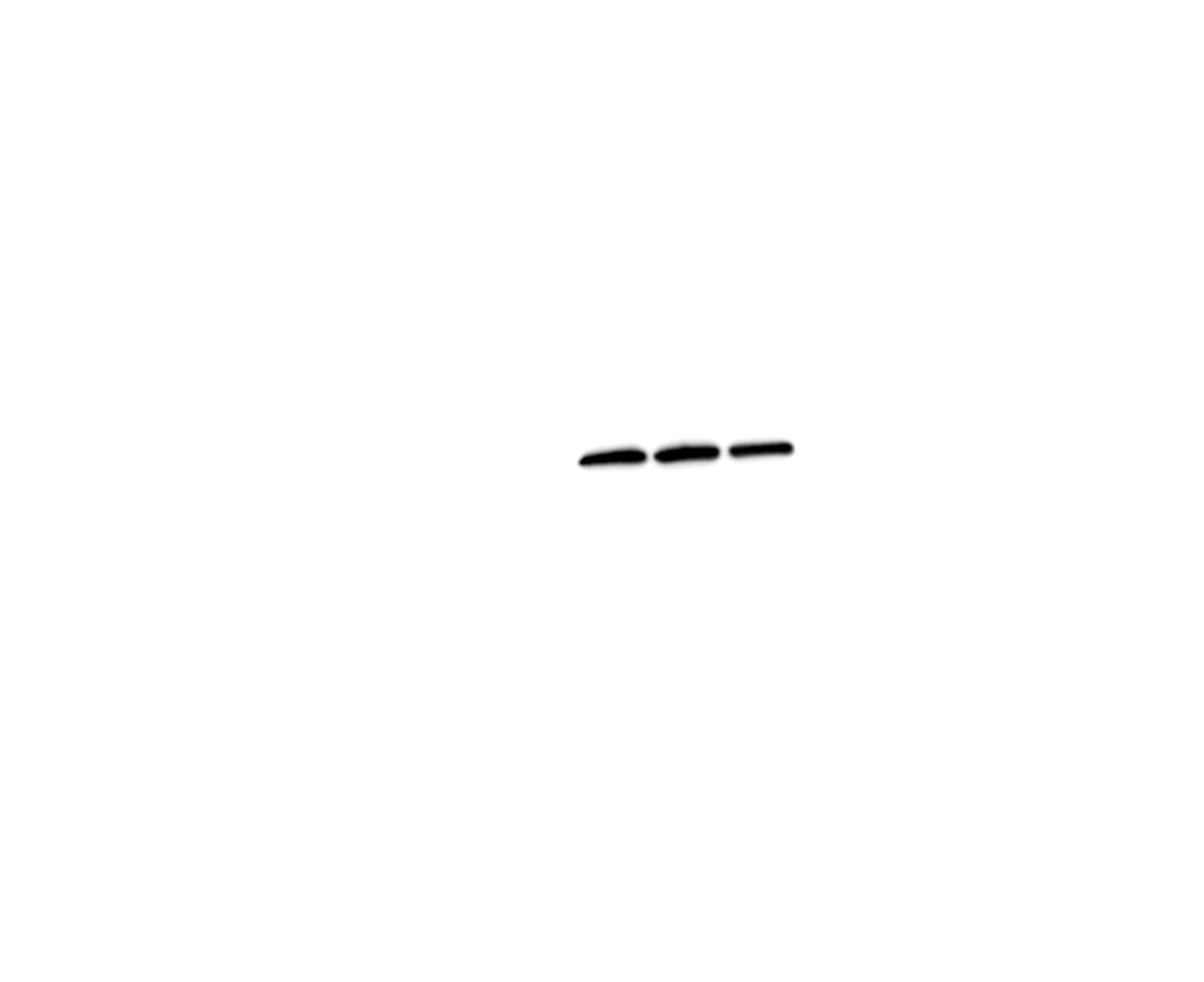 |
| **Figure 3C**-**ACTB** |
| 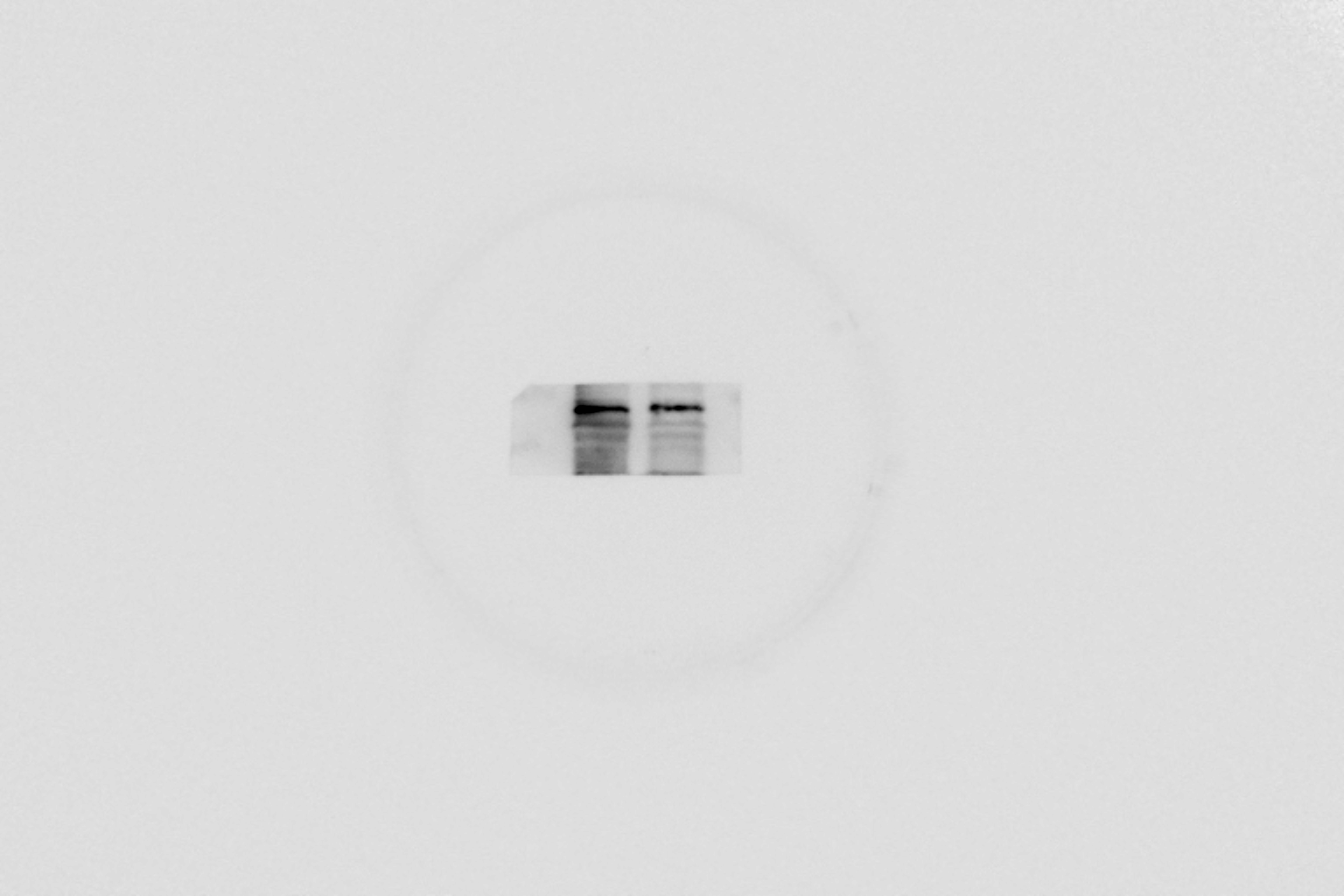 |
| **Figure 4A**-**p-AKT** |
| 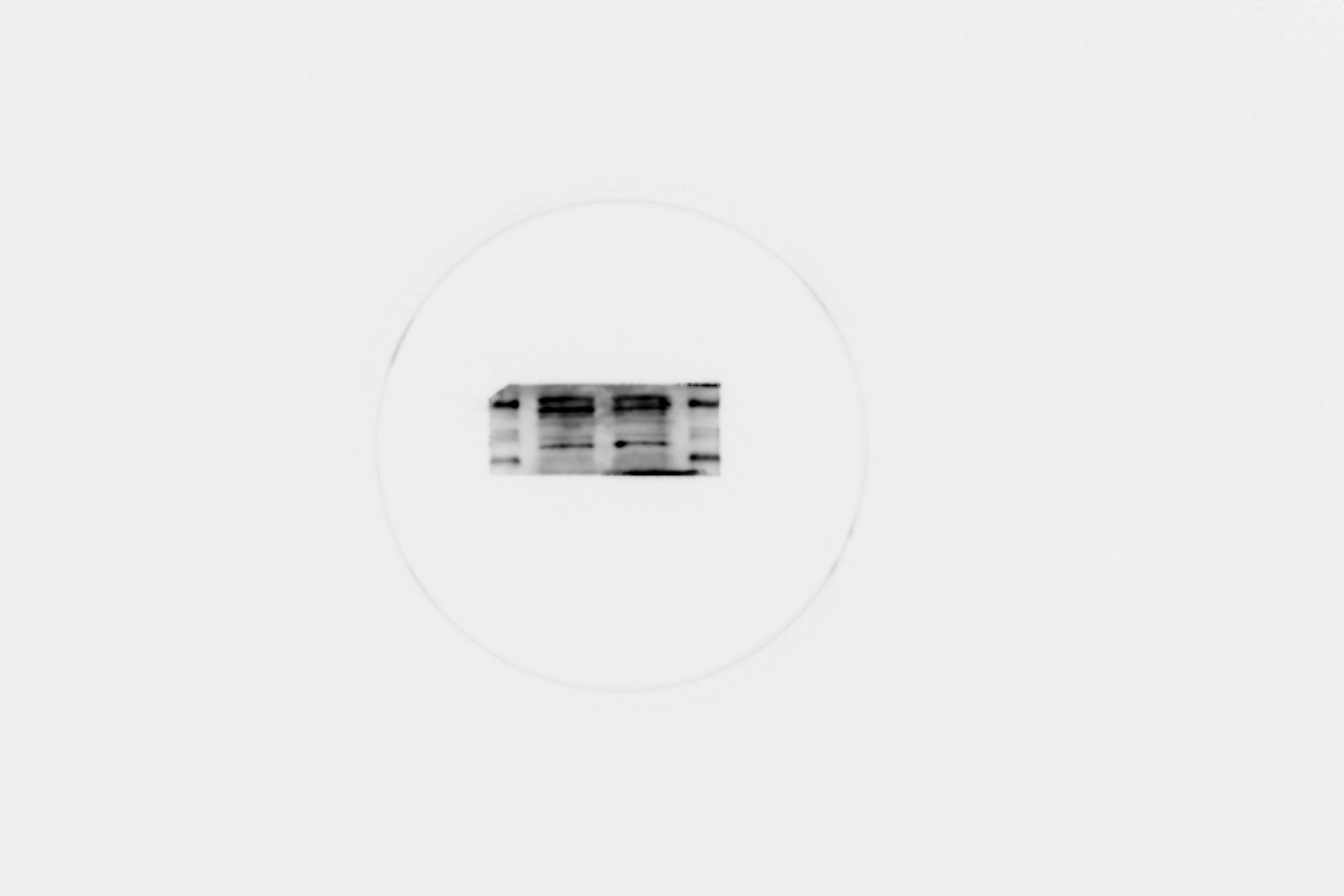 |
| **Figure 4A**-**AKT** |
| 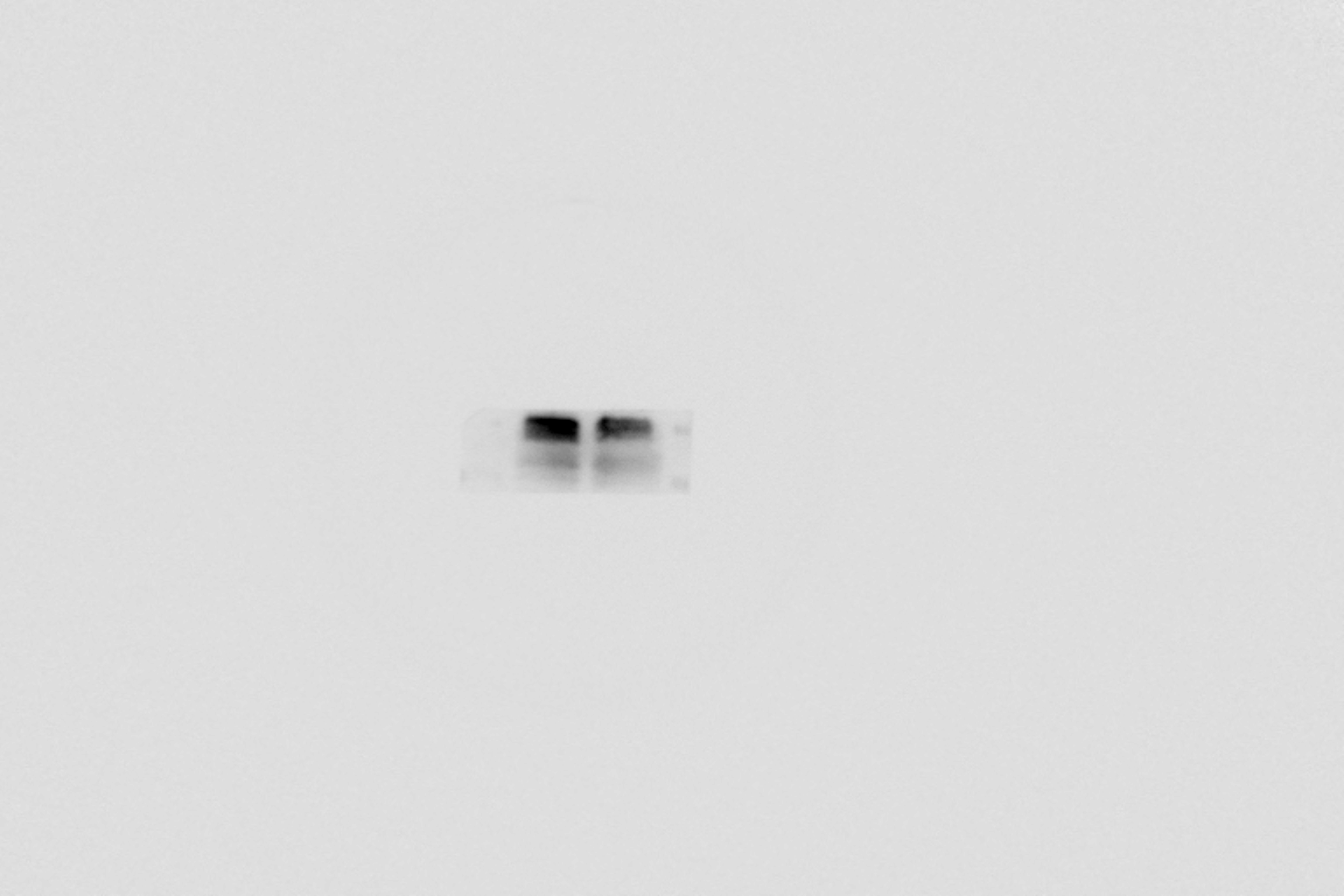 |
| **Figure 4A**-**p-EIF4EBP1** |
| 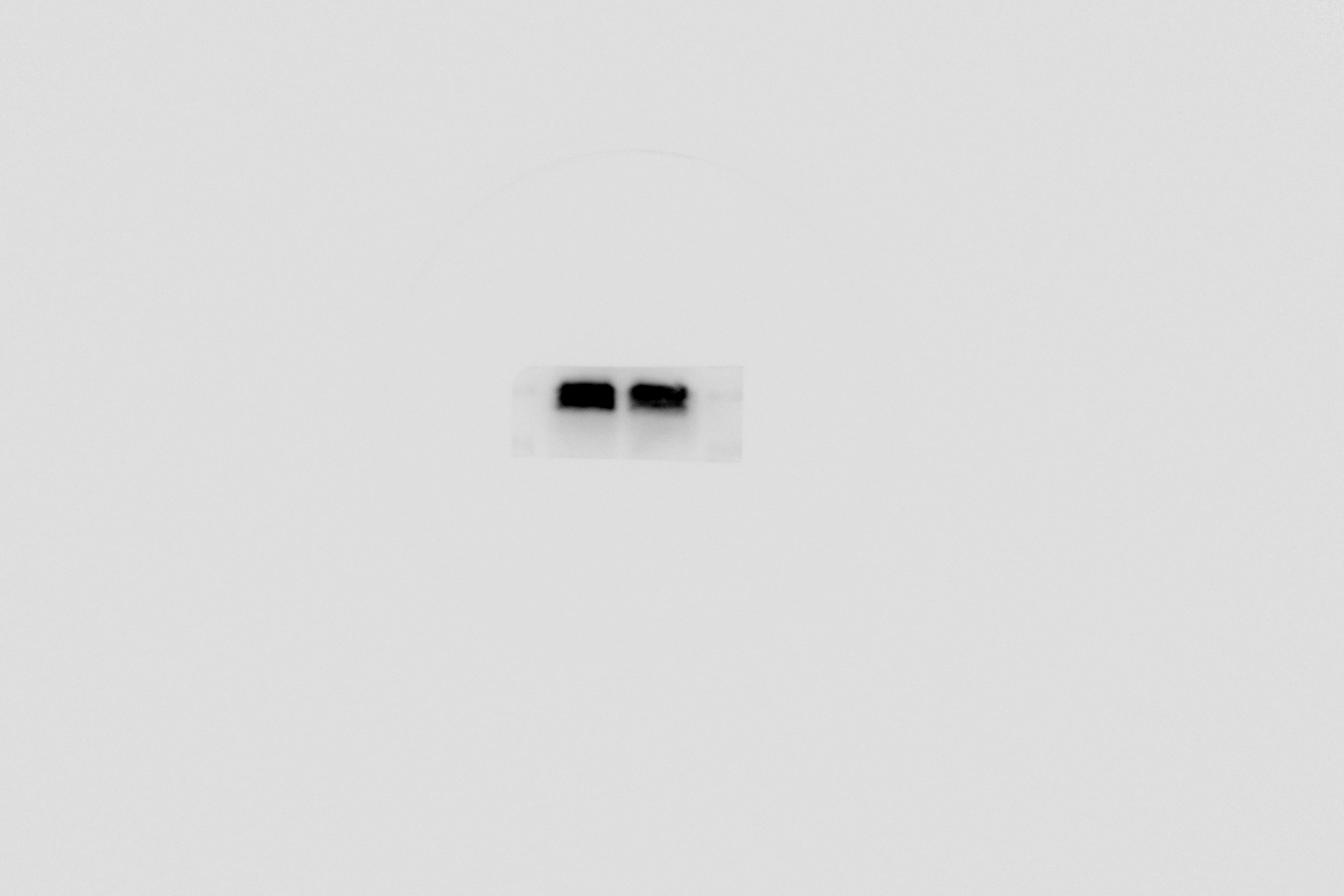 |
| **Figure 4A**-**eIF4EBP1** |
| 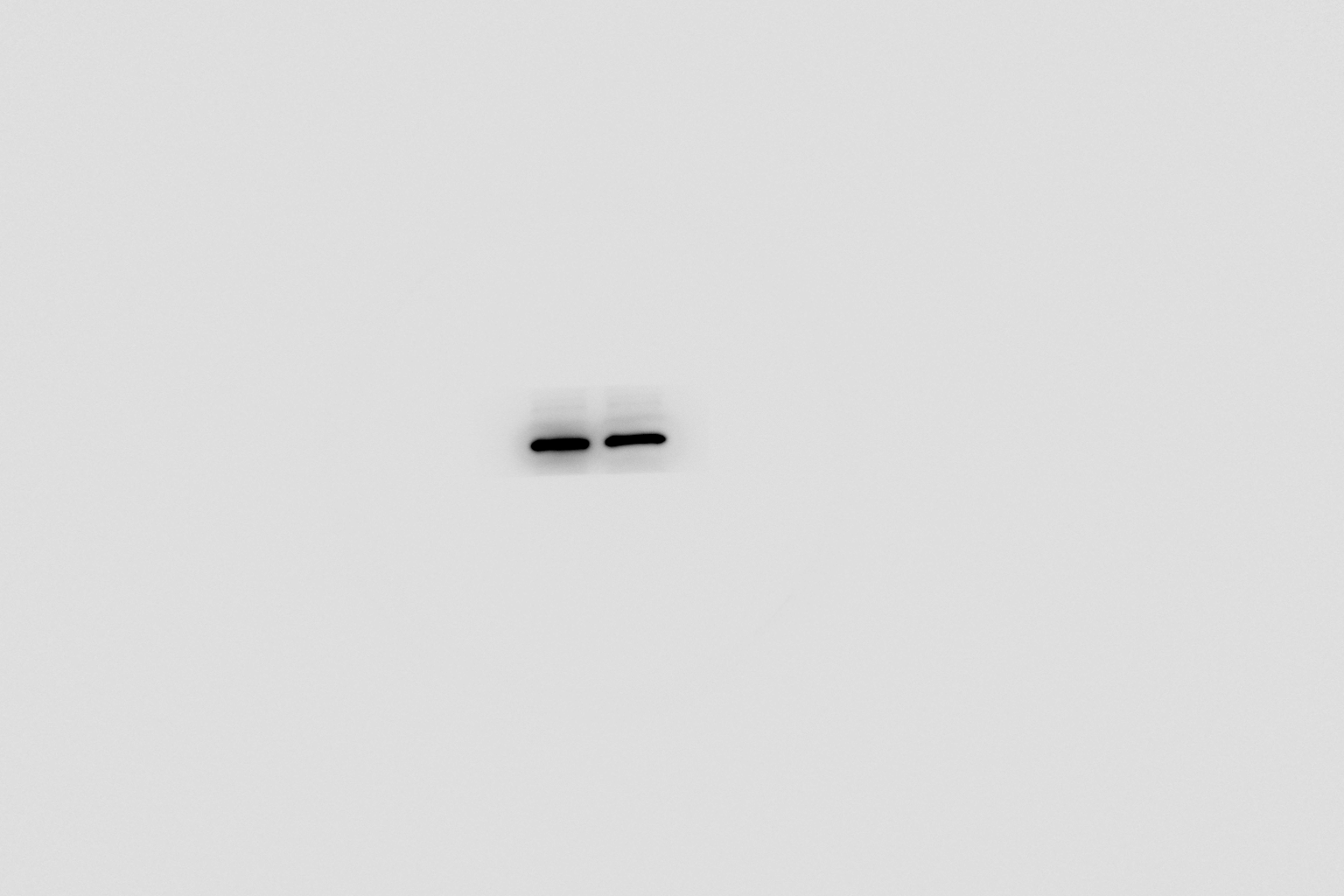 |
| **Figure 4A**-**ACTB** |
| 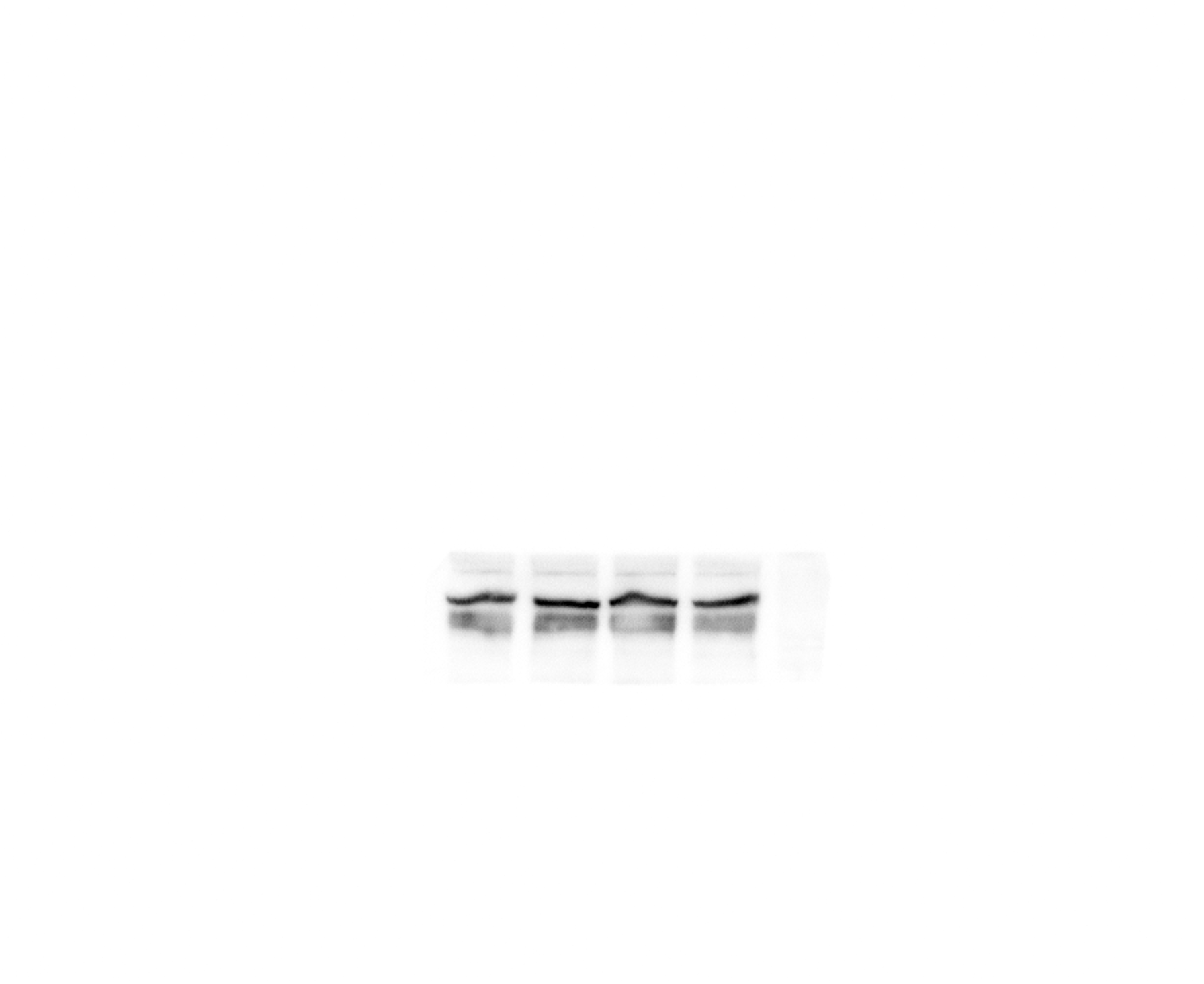 |
| **Figure 4B**-**MYOD** |
| 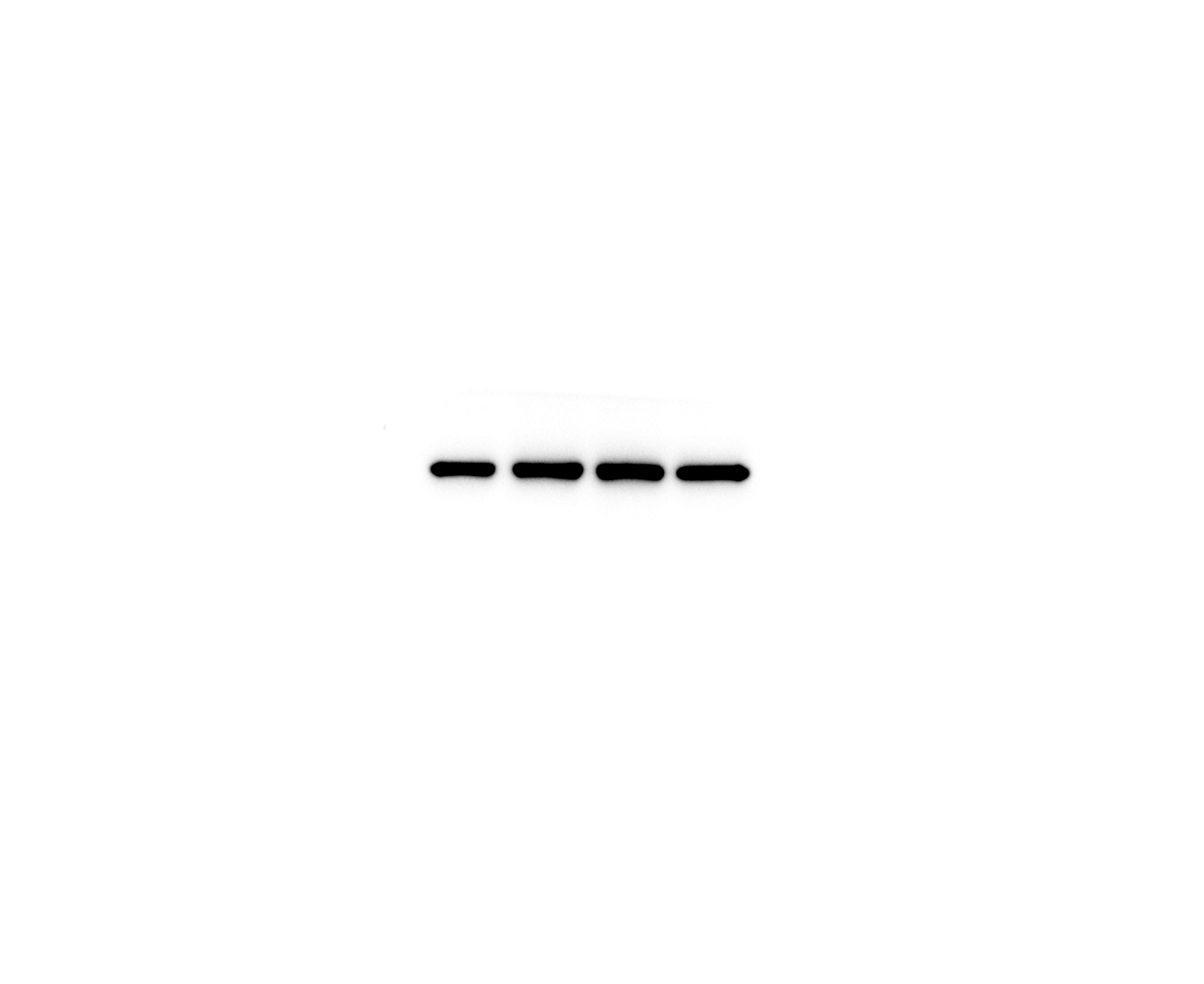 |
| **Figure 4B**-**ACTB** |
