# Supplementary material for: Retinoic acid signalling inhibits myogenesis by blocking MYOD translation in pig skeletal muscle cells
Source: Anim Biotechnol. 2024 May 16;35(1):2351973. doi: 10.1080/10495398.2024.2351973 (PMC12674307; doi:10.1080/10495398.2024.2351973)
Supplement: Supplemental Material [file LABT_A_2351973_SM4375.docx]

**Supplementary Data S3. Details of primers used for vector construction in this study.**

| Gene | Pairs Sequence (5′-3′) | qPCR  Amplification  length (bp) | Tm (°C) |
| --- | --- | --- | --- |
| RARγ | RARγ-CDS-F: ATGGCCACCAATAAGGAGCG  RARγ-CDS-R: TCAGGGGCCCTGGTCGGGGTG | 1374 | 62 |
| Amplification primers with homologous arms | RARγ- homo-F: GAACCGTCAGATCCGCTAGCGCTACCGGTCGCCACC ATGGCCACCAATAAGGAGCGA  RARγ- homo-R:  TGGCTGATTATGATCAGTTATCTAGATCCGGTGGATCCTCAGGGGCCCTGGTCGGG | 1452 | 62 |
