# Supplementary material for: Retinoic acid signalling inhibits myogenesis by blocking MYOD translation in pig skeletal muscle cells
Source: Anim Biotechnol. 2024 May 16;35(1):2351973. doi: 10.1080/10495398.2024.2351973 (PMC12674307; doi:10.1080/10495398.2024.2351973)
Supplement: Supplemental Material [file LABT_A_2351973_SM4374.docx]

**Supplementary Data S2. Details of primers used for qPCR in this study.**

| Gene  （Transcript ID） | Pairs Sequence (5′-3′) | qPCR Amplification length (bp) | Tm (°C) |
| --- | --- | --- | --- |
| RARγ  (ENSSSCT00045003481.1) | F: GGCTACCACTACGGGGTCAG  R: GGGGGCTCAGTTCATAGCTGTC | 259 | 60 |
| MYOD  （NM_001002824.1） | F: ACGACGGCACCTATTACAGC  R: AGCATTCTTCCCGGGCCG | 145 | 59 |
| MYOG  （NM_001012406.1） | F: TCAACCAGGAGGAGCGAGAC  R: GTGATGCTGTCCACGATGG | 211 | 59 |
| GAPDH  （NM_001206359.1） | F: GCTGCCCAGAACATCATCCC  R: AGGTCAGATCCACAACCGACAC | 136 | 60 |
| PAX7  （XM_021095460.1） | F: TCCAGCTACTCCGACAGCTT  R: TGCTCAGAATGCTCATCACC | 100 | 59 |
| KI67  （NM_001101827.1） | F: AGTCTGTAAGGAAAGCCACCC  R: ACAAAGCCCAAGCAGACAGG | 119 | 57 |
| BAX  （XM_003127290.5） | F: CCGAAATGTTTGCTGACG  R: AGCCGATCTCGAAGGAAGT | 154 | 54 |
