# Supplementary material for: Retinoic acid signalling inhibits myogenesis by blocking MYOD translation in pig skeletal muscle cells
Source: Anim Biotechnol. 2024 May 16;35(1):2351973. doi: 10.1080/10495398.2024.2351973 (PMC12674307; doi:10.1080/10495398.2024.2351973)
Supplement: Supplemental Material [file LABT_A_2351973_SM4373.docx]

**Supplementary Data S1.** **Abbreviations**

| ACTB | β-Actin |
| --- | --- |
| AKT | AKT kinase；protein kinase B |
| bFGF | basic fibroblast growth factor |
| CEE | Chicken embryo extract |
| DAPI | 4',6-diamidino-2-phenylindole |
| DMEM | Dulbecco’s modified Eagle’s medium |
| DMSO | Dimethyl sulfoxide |
| EdU | 5-ethynyl-2’-deoxyuridine |
| eIF4E | eukaryotic translation initiation factor 4E |
| eIF4EBP1 | eukaryotic translation initiation factor 4E binding protein 1 |
| GAPDH | glyceraldehyde-3-phosphate dehydrogenase |
| MEM | Minimum Essential Medium |
| NEAA | Non-Essential Amino Acids Solution |
| MYOD | myogenic differentiation 1 |
| MYOG | myogenin |
| MYHC | Myosin heavy chain |
| PAX7 | paired box 7 |
| PBS | Phosphate buffered saline |
| PFA | Paraformaldehyde |
| PMSF | Phenylmethanesulfonyl fluoride |
| pMuSCs | pig primary muscle stem cells |
| PPARδ | Peroxisome proliferator activated receptor delta |
| PVDF | polyvinylidene difluoride |
| RA | retinoic acid |
| RARγ | retinoic acid receptor gamma |
| RPMI 1640 | Roswell Park Memorial Institute 1640 medium |
| RT-qPCR | Real time quantititve Polymerase Chain Reaction |
